# Supplementary material for: Chromothripsis during telomere crisis is independent of NHEJ, and consistent with a replicative origin
Source: Genome Res. 2019 May;29(5):737–49. doi: 10.1101/gr.240705.118 (PMC6499312; doi:10.1101/gr.240705.118)
Supplement: Supplemental Material [file supp_gr.240705.118_Supplemental_file_1.zip › contigs/annotated_contigs/DB53/contig.6.DB53_length_1186_mean_cov_24.7757166948.docx]

**DB53_length_1186_mean_cov_24.7757166948**

```
GACCTTCCTGGATTTCTGCTGAAGCTGTGTTCTGGACCTCTCCAGGAGAGGCAGCAACGGTAATGATTAAGAGTTTAGGCTCCAGAATG
 >chr8:81609701-81610161 + E=1e-264
AGTTAGATAATGTGGCTTCAGATCCACTCTCTGCATTACCACCTGTGTGACCTTGGGCAAATTACTTAAACTCACTGTTTCCTTATCTG

TAAAAGAAGGATAAACATAGACTTGTTGAGAGGGTTAAATGAGAAAAGTATATGAGCACCAGCATGAGCCTCAGCCACAGTCAGAGTTC

ATGTTATCTCAGCTATTACTAACCTTCCAATTCCTGACTACATCCATAGCGTGTGTCCTCCGTGAGGGAGGAGAGCATTAGATGACTTC

ACTTGCTTTTCTTGTTCAGCACCAGCACTAGCAGTTGTGGATTTTCTTTTTGTAAGGCAGGTATATTATTTTTGATAATGAAAAGACTG

CTATAACTC|TGTAAG|TTAATAGAACAGTTTTTTTCCCCCAAATACTTCAGCAATAGAGAATGCTGAAAGAAAACAGAAAAATGGAAA
          >chr8:77875658-77875794 + E=9e-70
GAGCCCACTAAGAAGGACAAAGAGGAACCTGAGCTAGCAGGTTAGTTACATAGCTAAT|AACTCCTAAA|TGACTCTTTATTATATCAT
                                                                     >chr8:78552166-785
CTCTCCTAGTA|TT|TTGTATAGTATCGATCCATGACATATTAAAAATTTCTTAAAAATGACCCTTCACCACAGATGGCTTCAGATGTA
52198 - E=3e-07 >chr8:81611730-81611836 - E=1e-51 p=1e-02
CTGGTGTATATTGCCTTATGTATTATGATG|CTATCTT|TGGTGTATGTGAGTCACAATCTTGTC|CTAGCTCTAGCAATCATGAGAGT
                                      >chr17:68800479-68800505 + E=1e-03
AGAAGCA|TGCCTAACATGTTACCTCCCTTTTTCCTGCACAGTTCTCCAAAGAGGGATGTGTTCTCAGTCTTCACTTTCTTACCTTTTA
        >chr8:81035980-81036343 - E=3e-203
GTCATGTTCAGCTCAGGTAATTAGGCTTTTCCTTTGTGAGTTTCCTGACATGCTGTCGCTGATAATCACCAGTAACCACCTGCTCGCCT

TTCCTTCCTTACTAGACATTCTGTTGCACTGGACGCTGTTGTCCATTCCTTTTCTTGAAACTCTATTTCCAAATCTTTCCAGATGTTGA

ACTCTCCCAGTGTTTTTCATACCTCTTTGACCAGTACCAAGAATGTAGAACCAAGACTACATTCTTGGTCCATCCTTTATGACAGTTTC

CCTGCCTCTCTGTGC|ACTTCTTCATGGACGCCATACATT
```
